# Direct-Acting Antivirals and Host-Targeting Approaches against Enterovirus B Infections: Recent Advances

**DOI:** 10.3390/ph16020203

**Published:** 2023-01-29

**Authors:** Chiara Tammaro, Michela Guida, Federico Appetecchia, Mariangela Biava, Sara Consalvi, Giovanna Poce

**Affiliations:** Department of Chemistry and Technologies of Drug, Sapienza University of Rome, Piazzale A. Moro, 5, 00185 Rome, Italy

**Keywords:** Enterovirus-B, Coxsackievirus, Enterovirus-B inhibitors, drug discovery, antivirals

## Abstract

Enterovirus B (EV-B)-related diseases, which can be life threatening in high-risk populations, have been recognized as a serious health problem, but their clinical treatment is largely supportive, and no selective antivirals are available on the market. As their clinical relevance has become more serious, efforts in the field of anti-EV-B inhibitors have greatly increased and many potential antivirals with very high selectivity indexes and promising in vitro activities have been discovered. The scope of this review encompasses recent advances in the discovery of new compounds with anti-viral activity against EV-B, as well as further progress in repurposing drugs to treat these infections. Current progress and future perspectives in drug discovery against EV-Bs are briefly discussed and existing gaps are spotlighted.

## 1. Introduction

### 1.1. Enterovirus Classification and Pathogenesis

Enteroviruses (EV) are small non-enveloped viruses with a positive-sense single-stranded RNA genome. EV-related diseases affect millions of humans and mammalians yearly and are a serious health problem since the treatment of enteroviral infection is mainly supportive and no selective antivirals are commercially available [1]. The genus EV belongs to the Picornaviridae family and is made up of 15 species (12 EVs, 3 rhinoviruses (RV)), seven of which affect humans: four EVs A-D and three RVs A-C. Human EV species comprise over 100 serotypes, and this number is growing [2]. Among EV species, EV-B represents the second largest, with 64 serotypes (7 CV, 29 echoviruses, 28 EV). While successful vaccination programs could eradicate the well-characterized serotype poliovirus (EV-C), in the last decades, in the USA, China and South-East Asia, various outbreaks of non-polio EVs (e.g., EV-A71, EV-D68, Coxsackievirus (CV)-A16) have been of serious concern [1]. Clinically, EVs can cause illnesses which range from mild (the common cold, non-specific febrile illness, hand foot and mouth disease) to severe (respiratory illnesses, myocarditis, acute flaccid myelitis, encephalitis, pancreatitis, and paralysis) which can turn chronic in the immunodeficient and even be fatal [3]. EV-B can also contribute to a wide range of serious acute and chronic infections such as type 1 diabetes mellitus (T1DM) [4]. Faecal–oral and respiratory are the main routes of transmission. After a primary replication in the upper respiratory tract and intestine, EV-B can reach the lymph nodes and may induce secondary infections through the bloodstream [3,5].

### 1.2. Life Cycle of Enteroviruses

EVs share a general structure: they are non-enveloped viruses with icosahedral capsids containing a positive sense, single-stranded RNA genome. The EV life cycle starts with the binding to its receptor, followed by endocytic uptake in the cells. Once in the cell, the genome, covalently linked to the viral protein (VP) 3B, is released from the viral capsid (uncoating) and from the endosome to the cytoplasm to start translation. This affords a polyprotein which is in turn processed into four structural capsid proteins (VP1-4) and seven nonstructural proteins involved in replication (2A, 2B, 2C, 3A, 3B, 3C and 3D). The latter occurs on membrane replication organelles (ROs) and is aided by two host proteins: PI4KB (Phosphatidylinositol 4-Kinase Beta), which synthesizes phosphatidylinositol-4-phosphate, (PtdIns4P), and oxysterol- binding protein (OSBP), which recruits cholesterol. At this point, the synthesized RNA can be assembled into new virions or undergo a second round of replication [3]. 

### 1.3. Antivirals against Enterovirus Infections

**Figure 1:**
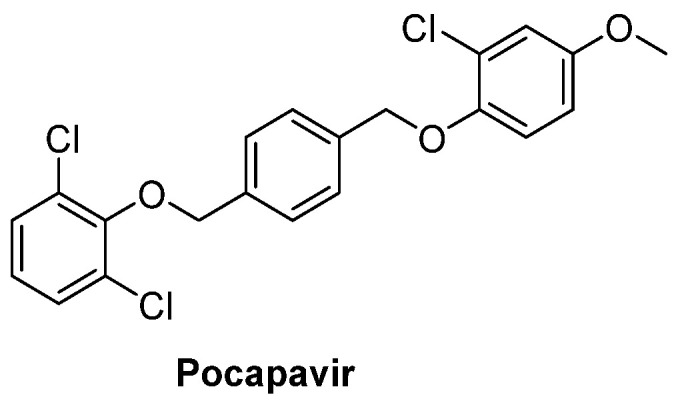
Chemical structure of the capsid binder pocapavir.

The identification of the key and pan-targets shared by these viruses is important to block viral invasions and/or replication [6]. The high recombination frequency in EVs corresponds to a large increase in the spread of new EV serotypes, hampering the development of new effective vaccines to control newborn infective agents. Accordingly, research has focused on broad-spectrum anti-viral drug projects. Antivirals can target either host-proteins or viral proteins, which have limited side effects but are more likely to induce resistance. Research has traditionally focused on the development of capsid binders and four drugs are currently under clinical investigation for the treatment of EV-infections: pleconaril, disoxaril, pirodavir, vapendavir and pocapavir [1,7]. Pocapavir (Figure 1) passed Phase I clinical trials and is currently undergoing Phase2a against poliovirus infections, but is available for compassionate use [8] and neonatal emergency for the treatment of severe EV-B infections [9,10]. Parallel research is focusing on the development of non-structural protein inhibitors such as protease, 3D^pol^ and 2C ATPase inhibitors, leading to promising compounds active against a wide range of EVs. For these targets, drug repurposing promises a time- and cost-efficient approach. Finally, a different strategy towards broader antiviral agents, less prone to induce resistance, is to target host factors involved in viral replication, but this approach could have negative side effects [11]. 

This review encompasses the recent advances in the discovery of new compounds with anti-viral activity against EV-B as well as the progress made with repurposed drugs to treat these infections. A brief introduction on the role of the selected targets is described and then followed by structure-activity relationship (SAR) studies and medicinal chemistry optimization of the active compounds. Current and future progress and perspectives in drug discovery against EV-Bs are briefly discussed and existing gaps are spotlighted.

## 2. Direct-Acting Antivirals

### 2.1. Capsid Binders

EVs share similar structures: their capsid consists of 60 identical protomers and each is composed of four viral capsid proteins, VP1, VP2, VP3 and VP4. Together, they form the icosahedral shell that contains the viral genome [3]. VP1-3 shape the capsid surface, while VP4 is in the inner surface of the capsid.

The external surface is characterized by star-shaped protuberances made of: (i) five VP1; (ii) circular depressions (called canyons) formed by VP1 and VP2-3 encircling the fivefold axis of symmetry; (iii) a prominence formed by the VP2 loop; (iv) a knob formed by the VP3 loop; (v) a twofold depression and (vi) the VP1 hydrophobic pockets bound by lipid molecules known as “pocket factors” [1]. The presence of several loops, accessible to the host immune system, and their diversity lead to a high antigenic diversity. The canyon, instead, serves as a receptor binding site. Capsid binders are among the most studied EV inhibitors. Three different regions on the EV capsid have been identified as possible targets for drug development. The first is the VP1 hydrophobic pocket, which is usually occupied by the so-called pocket factor (a lipid molecule); the antiviral compound displaces it and binds to the pocket, stabilizing the capsid in a rigid conformation. The second region is the VP1-VP3 interprotomer interface of the viral capsid. A third possible target is the fivefold axis of the capsid, which is responsible for viral attachment to host cell receptors and in many EV, including some of the EV-B members, has a positively charged fivefold axis. The axis is responsible for viral attachment to host cell receptors; however, as reported by Ren et al., suramin, which interacts with the fivefold axis and is active against many EV, did not show activity against EV-B, with the exception of CV-A9 [12]. Recently, Reshamwala et al. [13] reported that the polyphenols epigallocatechin gallate and resveratrol have a strong antiviral effect and are able to simultaneously bind multiple sites on the viral surface, reducing binding to host cells, inducing aggregation and preventing RNA release.

#### 2.1.1. Antivirals Targeting the VP1 Hydrophobic Pocket

**Figure 2:**
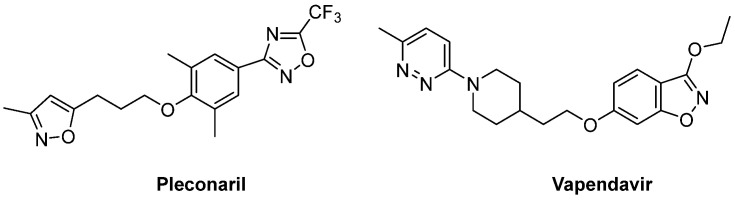
Chemical structures of the two capsid inhibitors pleconaril and vapendavir.

The VP1 hydrophobic pocket is the most studied target for anti-enteroviral compounds and is used to identify variant strains and new EVs. Pocket binders usually inhibit EV-B infectivity with IC_50_—half maximal inhibitory concentration—or EC_50_—half maximal effective concentration—in the µM range. Despite promising in vitro results, the two well-known pocket binders pleconaril and vapendavir (Figure 2), have failed the clinical trials due to their low efficacy and several side effects. Nevertheless, their discovery raised interest in the search for analogues [14,15]. 

**Figure 3:**
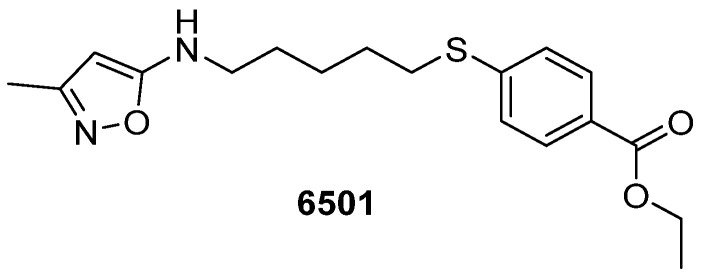
Chemical structure of compound **6501**.

Bernard et al. studied CV-B3 and echovirus inhibitors and found that compound **6501** (Figure 3) was active with an EC_50_ of 11 µM [14]. 

All but one analogue exhibited a positive cell protective effect in the echovirus assay, with EC_50_s ranging from 0.3 to 108 µM, and selectivity indexes (SIs) ranging from 3 to 1524. SAR studies highlighted the influence of the aliphatic chain on antiviral activity: the sulphur atom was pivotal for the activity against CV-B3, while the presence of an amino group in the aliphatic chain increased anti-echoviral activity [14].

**Figure 4:**
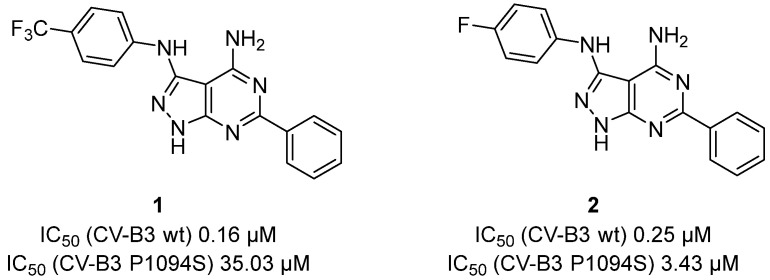
Chemical structures and anti-viral activities of compounds **1** and **2**.

Makarov et al. developed a series of pyrazolo [3,4-d]pyrimidines active against pleconaril-resistant variants of CV-B3 [16]. Compound **1** (Figure 4) showed good physicochemical, antiviral, and pharmacokinetic properties. It was also effective in a model of CVB3-induced chronic myocarditis in NMRI mice, decreasing inflammation and tissue damage in the pancreas and heart when administered 1 h, 1 day or 3 days post-infection. Despite the promising in vitro results, compound **2** (Figure 4), was almost inactive in vivo due to the formation of less active hydroxylated metabolites. Notably, the broad-spectrum antiviral activity of compound **1** overcomes resistance of known capsid inhibitors and its good oral availability makes it a promising drug candidate [16].

**Figure 5:**
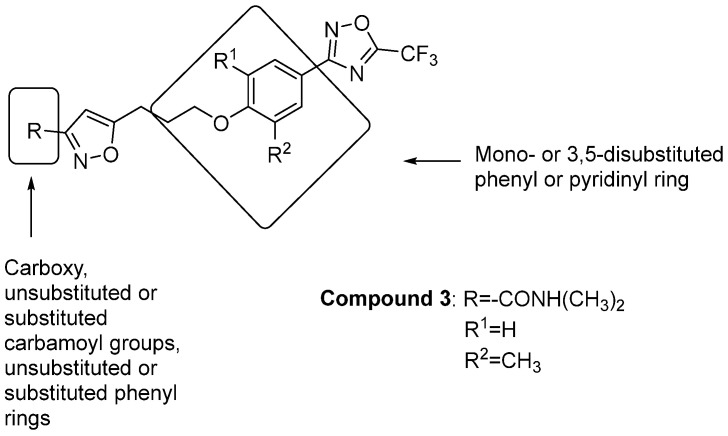
SAR studies on pleconaril analogues and chemical structure of compound **3**.

A very interesting contribution to the field of anti-EV binding the VP1 hydrophobic pocket comes from Egorova et al. [15], who studied the influence of substituents at position three of pleconaril on the inhibition activity against both pleconaril-sensitive and -resistant EV. They first evaluated the influence of the isoxazole ring substituents, obtaining IC_50_ values comparable to or even lower than pleconaril. SAR analysis showed the crucial role played by the N,N-dimethylcarbamoyl moiety on the isoxazole ring for antiviral activity against pleconaril resistant mutants (Figure 5). In particular, compound **3** proved to be the most active of the series against both pleconaril-resistant and pleconaril-sensitive EV with IC_50_ values of 0.02, 4.79, 0.01, 0.01, 2.76 (for CVs B3 97927 wt, I1207K, I1207M, I1207T and Nancy, respectively), 0.86, 5.25 and 0.09 μM (for RVs A2, B5 and B14, respectively). Compound **3** showed the potential for preclinical investigation since it also proved to be non-mutagenic and has a very high bioavailability after intragastric administration to mice [15].

**Figure 6:**
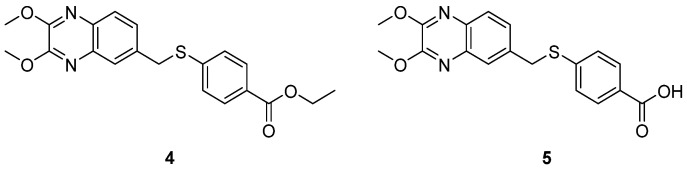
Chemical structures of compounds **4** and **5**.

Interestingly, a series of quinoxaline derivatives identified by Carta et al. showed outstanding and selective activities against CV-B5, a different CV-B serotype associated with myocarditis and encephalitis in immunocompromised children and central nervous system disorders in adults [17]. Compound **4** and its corresponding acid 5 (Figure 6) were the most active of the series, with an EC_50_ of 0.09 and 0.06 µM against CV-B5, respectively, and very high SIs (CC_50_ > 100 and > 65, respectively, in Vero-76 cells). A combination of experimental and computational studies revealed that derivative four blocks early stages of viral infection and prevents internalization without interfering with virus attachment. Molecular dynamics simulations suggested that VP-1 might be the target, and predicted multiple and high-energy interactions, but further experimental studies are needed to prove this hypothesis [17].

The activity of these broad-spectrum compounds was further evaluated against a panel of representative EVs (EV-71, of the EV-A species, CV-B4, CV-B3 and echovirus 9, and EV-D68, of the EV species B and D, respectively). Compounds **4** and **5** retained one-digit micromolar activities against both CV-B4 and CV-B3 but were inactive against the other EV species tested. This CV-selective activity could be partially explained by differences in the VP-1 protein sequences. Moreover, an RNA-binding motif analysis through an RNA-Binding Protein DataBase (RBPDB) aimed at revealing new targets in CVs, revealed that only CV-3 and CV-4 share the binding motif for SRSF13, a serine-arginine rich splicing factor involved in splicing events which might alter viral replication and be a potential innovative target for these compounds [18].

#### 2.1.2. Antivirals Targeting the VP1-VP3 Interprotomer Binding Pocket

**Figure 7:**
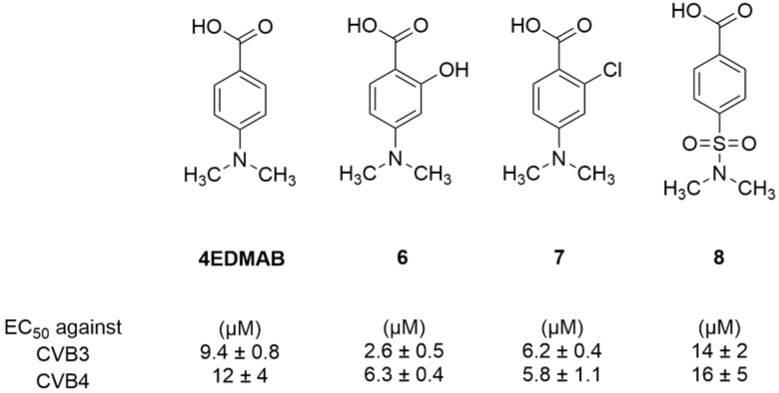
Chemical structures and activities against CVB3 and CVB4 of 4EDMAB and compounds **6**–**8**.

In 2017, Ma et al. identified a new target: a novel conserved site in VP1, close to the canyon but different from the hydrophobic pocket bound by the “classical” capsid binders. [19]. It was identified by screening a series of substituted benzoic acids highly selective against CV-B viruses and ineffective against other EVs, such as echovirus. A cell-based antiviral screening revealed that 4-dimethylamino benzoic acid (4EDMAB, Figure 6) was a selective inhibitor of CV-B3 replication (EC_50_ = 9.4 μM), and inactive against other EVs, including echovirus. The authors assessed antiviral activities of twenty-three analogues of 4EDMAB to study their SARs. The obtained data showed that the introduction of a hydroxyl group at the ortho position of the benzoic acid (compound **6**, Figure 7) led to an EC_50_ of 2.6 µM (3.6 fold more potent than 4EDMAB), while the introduction of a chlorine atom (compound **7**, Figure 7) at the same position, slightly improved the activity (EC_50_ = 6.2 µM). This proved that the introduction of substituents on the phenyl ring significantly affects the antiviral activity. Modifications of the dimethylamino group were detrimental: only compound **8** (Figure 7) maintained an antiviral activity against CV-B3, comparable to 4EDMAB) [19].

A time-of-addition assay and mutational and molecular modelling studies revealed that 4EDMAB blocks the early stages of the replication cycle and binds to a cavity of the viral capsid different from the hydrophobic pocket targeted by pleconaril and its analogues. The carboxylic group of 4EDMAB interacts with the Arg219 residue, consistent with the fact that esterification of the carboxylic group led to a loss of activity [19].

**Figure 8:**
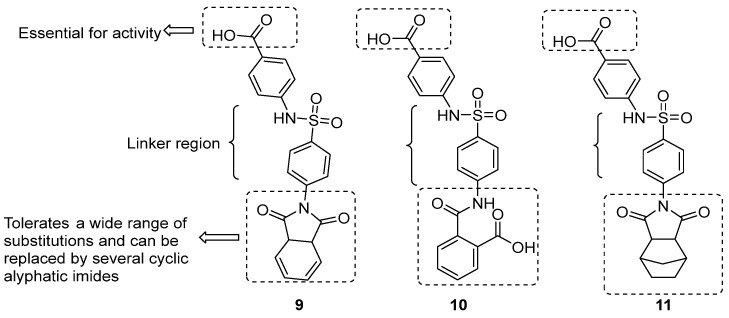
Chemical structures of compounds **9**–**11**.

In 2019, Abdelnabi et al. identified a benzene sulfonamide derivative (compound **9**, Figure 8) with a submicromolar activity against CV-B3 (EC_50_ 0.7 µM) without adverse effects on cells up to 296 µM. It also retained activity against CV-B1, B4, B5, and B6 and had a moderate effect against CV-A9. Structural studies revealed that compound **9** binds to a pocket formed by two VP1 and one VP3 units, at the interface of an interprotomer. The pocket seems to be conserved across the EV-B group, as shown from the analysis of 56 CVB3 amino acid sequences from GenBank, but the inhibition mechanism is not fully understood; it may be that the binding stabilized the conformation of the virion, resulting in an increase of the barrier energy and then to an inhibitory effect [20]. A very recent study has further explored the potential of this scaffold, affording two hits (compounds **10** and **11**, Figure 8) acting in the low micromolar concentration (IC_50_ = 4.29 and 4.22 µM, respectively). SAR studies revealed that the phtalimide region is amenable to modification, while the carboxyl moiety is essential for activity, suggesting that this should be primarily responsible for ligand-receptor binding [21].

### 2.2. Non-Structural Protein Inhibitors

#### 2.2.1. 3C Protease Inhibitors

**Figure 9:**
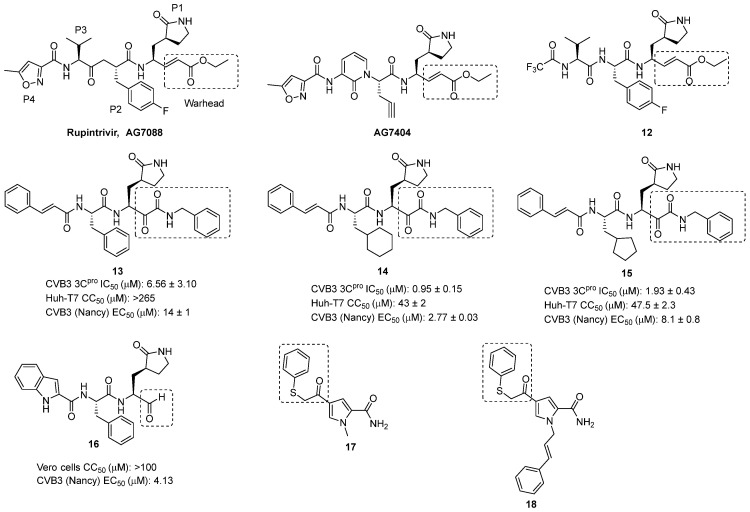
Chemical structures of rupintrivir, AG7404 and compounds **12**–**18** and biological activities of compounds **13**–**16**. Highlighted in the boxes are the main chemical motifs responsible for the interaction with protein 3C.

3C protease (3C^pro^) is a valuable target for the development of new antiviral compounds since it plays an important role in the viral replication cycle. Targeting 3C^pro^ also reduces off-target effects, as this protease is not present in human cells [11]. Rupintrivir, also known as AG7088 (Figure 9) and initially developed by Pfizer to cure the common cold, is a selective irreversible inhibitor of 3C^pro^ with broad-spectrum activities against EVs. Despite its poor oral bioavailability, it was selected for clinical trials for rhinovirus cold treatment, but they were stopped because rupintrivir did not reduce disease severity in naturally infected patients [22,23]. A recent study has shown a synergistic effect of rupintrivir and pleconaril against EV1-infected cells, suggesting that such combination should be further explored in clinical trials against EV1 and other EV infections [24]. The analogue AG7404 (Figure 9) was developed to overcome rupintrivir’s poor oral bioavailability. It was characterized by good in vitro activities and a satisfactory in vivo safety profile. It also showed an acceptable PK and safety profile in Phase I clinical trials, supporting further clinical investigation [23,25]. Two different assays were performed to detect the inhibitory potency of rupintrivir and AG7404 against CV-B3: a protease assay and a multicycle CPE-reduction assay. EC_50_ values confirmed the inhibitory activity of these compounds. The main chemical difference between AG7088 and AG7404 is a rigid pyridine in AG7404 in the place of the isopropyl chain of AG7088. Even though this rigid closure impairs the flexibility of the molecule, both compounds can interact with 3C^pro^ of CV-B3. The essential motif, ensuring the interaction of these peptidomimetic compounds with the target is the α,β-unsaturated ester, forms an irreversible covalent bond to a sulfhydryl group of a cysteine residue of 3C^pro^ [26,27].

The replacement of the 5-methylisoxazole ring of rupintrivir with a trifluoromethyl substituent led to compound **12** (Figure 9), which showed a micromolar inhibitory activity against echovirus 7, another serotype of EV-B, and had an EC_50_ (65 nM) close that of rupintrivir against EV-A71 (16 nM). Computer modeling studies compared the interactions between rupintrivir and compound **10** with 3C^pro^. They showed that the α,β-unsaturated ester directly interacts with the SH-cysteine of 3C, forming an irreversible bond and then inhibiting the protease activity, while the P4 residue is critical for membrane permeability but not for antiviral activity [28].

The common features shared by the enteroviral 3C^pro^ and the coronaviral main protease (M^pro^) inspired the structure-based design of a new class of broad-spectrum protease inhibitors targeting both coronaviruses and enteroviruses [29]. A series of peptidomimetic α-ketoamides was then developed to assess the feasibility of this approach. Extensive crystallographic studies of six inhibitors co-crystallized with three different proteases proved that the α-ketoamide warhead is more versatile and amenable to chemical modifications than Michael acceptors and aldehydes, as it has two hydrogen bond acceptors instead of one [30]. The first compound of this series (compound **13**, Figure 9) exhibited good potencies against both viral strains, retained a good cell activity and was generally non-toxic, with SIs > 10. Crystallographic studies revealed that the α-ketocarbon is covalently bound to the active-site Cys, resulting in a thioemiketal in the *S* configuration in the CV-B3 3C^pro^ complex. Structure-based optimization of compound 13 focused on diversifying the size and flexibility of the P2 substituents. The replacement of the phenyl moiety with an aliphatic cycle dramatically improved the activity and afforded two improved hits (compounds **14** and **15**, Figure 9), with satisfactory activities against isolated proteases with outstanding antiviral activities in Vero cells and acceptable cytotoxicities (Figure 9). Apart from identifying two novel antivirals, this study also highlighted the main differences between the two proteases, demonstrating the great potential of a structure-based approach in the search for novel broad-spectrum antivirals.

The chemical scaffold of this series of peptidomimetics was further explored by changing the α-ketoamide warhead with an aldehyde. These compounds were tested against a panel of EVs and coronaviruses. Despite its outstanding anti-EV-68 and anti CV-A21 activities, compound **16** potency (Figure 9) against CV-B3 was relatively weak, which could be due to differences in the substrate binding pocket [31].

A structurally non-related class of covalent inhibitors was identified by high-throughput virtual screening using 3D pharmacophores to identify innovative warheads and covalently binding fragments. The screening identified several fragment hits, and the C5 phenylthiomethyl ketone 17 (Figure 9) was selected for synthetic fragment growing, yielding compound **18** (Figure 9). A combination of mass spectrometry and enzymatic assays validated virtual hits and revealed two possible binding modes: a reversible, through the formation of a thioemyketal, or an irreversible, mediated by a thioether. This study identified a new class of 3C^pro^ inhibitors characterized by a covalently binding moiety for irreversible inactivation for further hit-to-lead optimization [32].

Even though covalent inhibition can afford strong irreversible inhibitors, their electrophilic nature can increase the risk of off-target effects. A safer approach could be the allosteric inhibition. The screening of 2000 compounds against CV-B3 3C^pro^ afforded the hit benserazide, a commercial drug which has no electrophilic sites for covalent inhibition and is used against Parkinson’s disease. Kinetic studies displayed non-competitive interactions consistent with a reversible allosteric inhibition mechanism, and this compound was selected for further hit optimization studies. SAR expansion of this scaffold afforded compounds with an increased affinity for the protease, and improved activities with IC_50_ in the submicromolar range. For many of them, however, this did not translate into antiviral efficacies, due perhaps to permeability issues [33].

#### 2.2.2. 2C Protease Inhibitors

**Figure 10:**
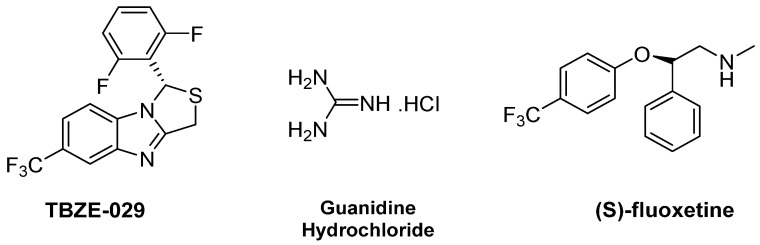
Chemical structure of TBZE-029, GuaHCl and *(S)*-fluoxetine.

The viral protein 2C is one of the most common enteroviral conserved proteins [34]. It belongs to the family of helicase proteins with an ATPase domain and exploits the energy from ATP hydrolysis to remodel RNA or DNA. 2C is characterized by three main motifs: Walker A, Walker B and motif C. These elements are involved in the formation of the ATPase domain responsible for ATP hydrolysis. The N-terminal domain of 2C shows a membrane-binding function, while the C-terminal can form oligomeric species. Indeed, 2C requires oligomerization to act and assist the replication process [35]. TBZE-029 (Figure 10) was the first identified inhibitor of protein 2C and belongs to a series of 2,6-dihalophenyl-substituted 1H,3H-thiazolo[3,4-a]benzimidazoles. It completely inhibits the accumulation of positive-stranded RNA at a concentration of 80 μM and reduces the accumulation of RNA by up to 40% at 3.3 μM. A time-of-drug-addition assay showed that TBZE-029 acts at a viral RNA replication stage. Genotyping of TBZE-029-resistant CV-B3 identified three amino acid mutations at positions 224, 227, and 229 in protein 2C. To confirm that protein 2C was the target of TBZE-029, a TBZE-029-resistant CV-B3 strain was generated and then tested for cross-resistance with other 2C inhibitors, such as guanidine hydrochloride (GuaHCl). The results confirmed that GuaHCl and TBZE-029 shared the same mechanism of inhibition without affecting 2C ATPase activity [36].

Commercial drug phenotypic screening is a growing trend for the discovery of novel antivirals. The repositioning of marketed drugs, which have already passed clinical trials, can guarantee their safety and allow for time and money-saving [37,38,39,40,41]. A molecular screening of a National Institutes of Health (NIH) chemical library against EVs identified fluoxetine (Figure 10), an FDA approved drug, for the treatment of major depression and anxiety disorders [42]. Further studies showed that the EC_50_ of fluoxetine against CV-B3 is 3.36 μM and mode-of-action studies have shed light on the role of the chirality for the activity. While the racemic mixture showed an EC_50_ of 3.2 μM, the *(S)*-enantiomer proved to be more active with an EC_50_ of 0.4 μM; surprisingly, the *(R)*-enantiomer did not show any effect against CV-B3. Through a homology model of 2C from CV-B3, the authors predicted that the 4-(trifluoromethyl)phenyl ring of *(S)*-fluoxetine lies in the deep hydrophobic pocket close to residues L126, L178, V187, F190, L238, I227, A229, and F242 [34]. Hurdiss et al. investigated its mode of action by generating the crystal structure of the soluble fragment of CV-B3 2C in complex with fluoxetine. Cryo-electron microscopy analysis demonstrated that fluoxetine binds the 2C protein in an exameric, stabilized state which results in an “open conformation” which could not assist the replication process. Since this conformational state of 2C implies that the protein cannot express ATPase activity, fluoxetine can be considered a 2C ATPase dose-dependent inhibitor. [35].

**Figure 11:**
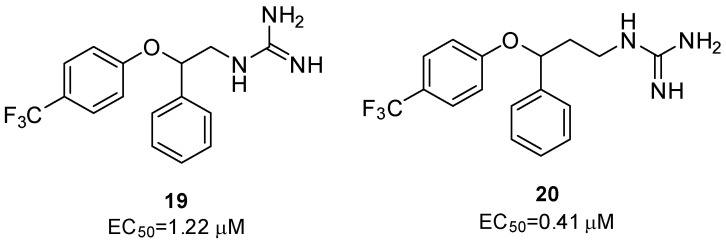
Chemical structures and EC_50_ of compounds **19** and **20**.

Since this data supported the potential use of fluoxetine as an antiviral, several research groups have synthesized fluoxetine analogues in the search for more potent compounds. Manganaro et al. investigated the importance of the *p*–CF_3_ substituent by moving it to the ortho or meta positions, or by introducing a second substituent [43]. The authors also designed fluoxetine analogues, replacing the methylamine group with the pan-enterovirus inhibitor GuaHCl or an acetyl group, to explore the need of a basic nitrogen. While moving the -CF_3_ substituent or introducing a second one on the ring proved detrimental, changes on the amino motif, especially the introduction of a guanidine group, retained antiviral activities (compounds **19** and **20**, Figure 11). Further studies on the *(R)* and *(S)* enantiomers of compound **18** confirmed the importance of the stereochemistry. As with fluoxetine, the (*S*) enantiomer showed improved antiviral activity (EC_50_ = 0.19 μM) compared to both the (*R*) enantiomer (EC_50_ = >30 μM) and the racemic mixture of 20 (EC_50_ = 0.41 μM) [43].

**Figure 12:**
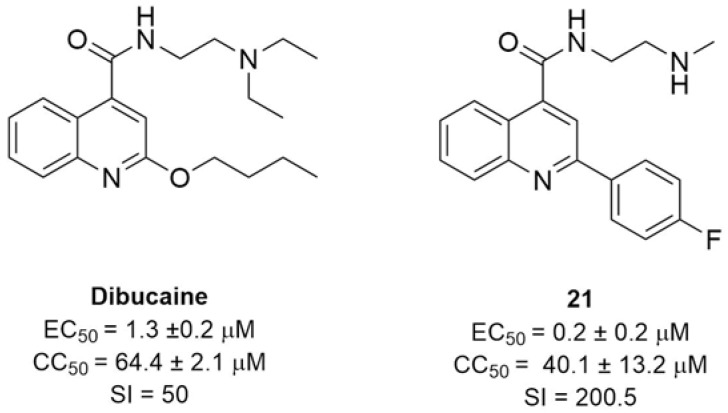
Chemical structures, EC_50_, CC_50_ and SIs against CV-B3 in Vero cells of dibucaine and its analogue **21**.

An independent screen yielded four other FDA-approved drugs: pirlindole, dibucaine, zuclopenthixol and formoterol, inhibiting CV-B3 replication more than 20-fold [44]. Despite their different chemical structures, they were all active against protein 2C-resistant strains and a direct binding was observed between dibucaine and protein 2C [44]. Since 2C is relatively conserved among non-polio enteroviruses, 2C inhibitors should have broad-spectrum antiviral activity against non-polio enteroviruses. Based on this hypothesis, in 2020, Musharrafieh et al. synthesized 43 quinoline analogues of dibucaine and profiled their antiviral activities against additional EV-D68, EV-A71, and CV-B3 viruses to obtain broader antivirals. Promising candidates were also tested to determine mouse microsomal stability. Compound **21** (Figure 12) was selected as a promising lead candidate and showed high antiviral activity and low cytotoxicity. Further studies highlighted that compound **21** directly binds 2C and stabilizes the structure of the protein in a dose-dependent manner, so as to impair its role in the replication process. Essential elements in its structure are the terminal monoalkylamine, which is responsible for half-life improvement, and a fluorine atom at the para position of the benzene ring, which improves the microsomal stability [45].

**Figure 13:**
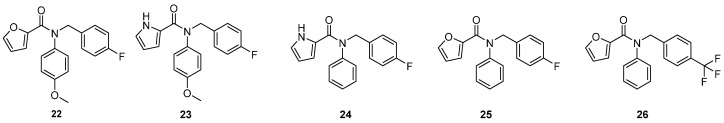
Chemical structures of compounds **22**–**26**.

Three years after the discovery of fluoxetine as a potential antiviral, the same research group extended the study by screening a large small-molecule library (85,585 compounds) and identified a wide array of chemical scaffolds interfering with viral replication [46]. Based on their drug-likeness, 12 compounds were selected for further examination and resistant mutants were generated to identify their potential target and mechanism of action. Interestingly, sequencing of the compound-resistant mutants revealed that most of the mutations were located at the 2C protein. The compounds identified were heterocycles, with accessible chemical modification allowing the generation of a facile SAR. Therefore, two research groups conducted independent hit-optimization studies. Bauer et al. developed a class of new 2C-targeting antivirals based on the backbone of a N-(4-fluorobenzyl)-N-(4-methoxyphenyl)furan-2-carboxamide (compound **22**, Figure 13) [47]. Several derivatives were prepared with the aim of: *(i)* replacing the furan ring with different heteroaromatic moieties and diverse heterocyclic/aliphatic groups; *(ii)* exploring the role of the amide bond through its replacement with either a sulfonamide or a methylene bridge; *(iii)* introducing different substituents on the 4-position on both rings of the N-benzylaniline moiety. SAR analysis highlighted that only the furan amide was essential for antiviral activity, but different modifications are possible. In particular, the two furan analogues **23** and **24** and the N-benzylaniline compounds **25** and **26** showed a broad-spectrum of EV and RV activity and, unlike fluoxetine, were not neuroactive.

**Figure 14:**
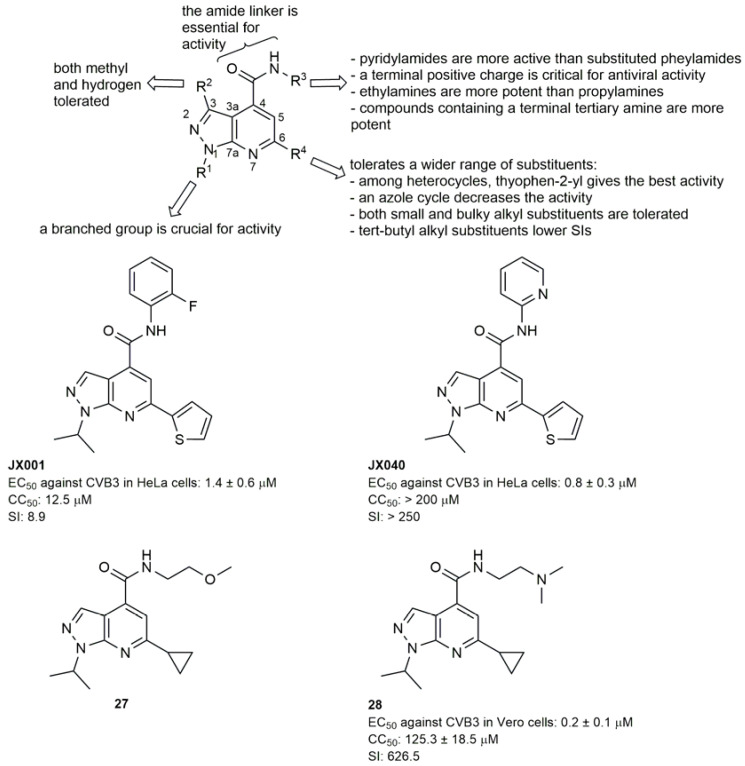
SAR analysis of the 1H-pyrazolo[3,4-b]pyridine scaffold and chemical structures and biological activities of compounds JX001, JX040, **27** and **28**.

Xing et al. optimized instead the pyrazolopyridine carboxamide series and built up an extended SAR study using JX001 as a reference compound (Figure 14). The development of a straightforward synthetic route allowed the synthesis of several analogues obtained by systematically modifying positions N1, C6, C4, the linker unit, and the central heterocycle. Compounds were tested against representative EV-A, EV-B and EV-C serotypes (EV-A71, CV-B3 and PV-1, respectively), and JX040 (Figure 14) showed submicromolar activity against CV-B3 in HeLa cells and very high SIs (>250). Interestingly, the introduction of new heterocycle ring systems other than 1H-pyrazolo[3,4-b]pyridine, abrogated antiviral activities. Only pyrrolopyridine analogues retained activity both on EV-A71 and CV-B3, and could represent a structurally new class of antiviral compounds for further hit-to-lead optimization [48]. A structurally related pyrazolopirimidine analogue (compound **27**, Figure 14) was also identified through a cytopathic effect (CPE) assay against EV-D68. In the search for broader antivirals, this hit was also tested against EV-A71 and showed nanomolar activities and high SIs [49]. An extensive SAR study (Figure 14) was then initiated to improve antiviral activities and SIs, and several analogues were generated by decorating the 1H-pyrazolo[3,4-b]pyridine scaffold with different substituents. Compound **28** (Figure 14) was then selected as a chemical probe for mechanistic experiments using the representative EV-D68 US/MO/14-18947 strain as a model. Two mutations at the 2C protein were identified, and direct binding with the CV-B3 2C protein was demonstrated through a thermal shift assay, suggesting 2C as a common target for EV-D68, EV-A71, and CV-B3.

An innovative and alternative strategy to develop antiviral drugs is to design peptide compounds that can modulate protein-protein interactions [50]. In this context, Fang et al. synthetized a peptide compound, 2CL, based on the amino acid sequence of the EV-A71 2C C-terminal domain. 2CL impaired the oligomerization of EV-A71 2C and showed a strong in vivo antiviral activity in mice infected with EV-A71. Following the same strategy, they designed a second peptide, B-2CL, to simulate the structures of the α6 helix of EV-B 2C. For this purpose, they compared the amino acid sequences of 2CL in EV-As with those in EV-Bs and observed some differences in a few residues. Peptide B-2CL was tested on CVB3- or echovirus-infected rhabdomyosarcoma cells and it effectively inhibited the replication of both serotypes, with an IC_50_ of 2.29 or 0.38 µM, respectively [51].

#### 2.2.3. 3A Protein Inhibitors

3A is a non-structural protein that plays a major role in viral RNA synthesis and assembly by mediating the formation of replication organelles (RO) through the recruitment of host factors essential for replication [52]. It also plays a crucial role in evading host cell immune response by inhibiting the ER-to-Golgi transport, thus causing protein accumulation. Therefore, this protein represents a valuable target to develop anti-viral compounds [53].

**Figure 15:**
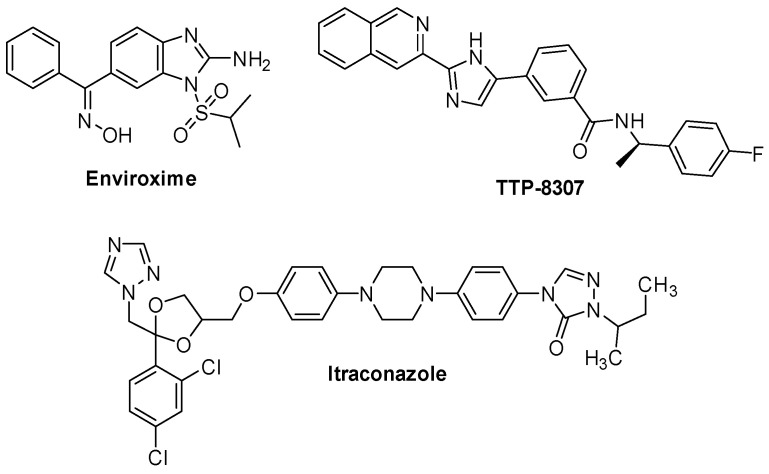
Chemical structures of enviroxime, TTP-8307, and itraconazole.

Enviroxime (Figure 15), a benzimidazole compound, is a broad-spectrum enterovirus antiviral targeting 3A [54]. It was evaluated in double-blind, placebo-controlled clinical trials in volunteers for RV infections; however, in spite of its potent antiviral activity, its development was halted due to gastrointestinal side effects and unfavorable pharmacokinetic profile [55].

TTP-8307 (Figure 15), identified as a broad-spectrum enterovirus inhibitor, was initially developed as a 3A inhibitor [56]. Indeed, single point mutations in the protein 3A of CVB3 generated a drug-resistant phenotype [56]. Further studies showed that these mutations harbored resistance against several other compounds targeting PI4KIIIβ and oxysterol-binding protein (OSBP). Albulescu et al. proved that TTP-8307 inhibits the lipid transfer function of OSBP in vitro [57]. Moreover, screening of an FDA-approved compound library of 1280 clinical compounds identified itraconazole (Figure 15), an antifungal triazole drug, as a broad-spectrum inhibitor of EVs. It inhibited EV-A71 replication in the low-micromolar range and was active against CVB3 with an EC_50_ ranging from 0.12 to 0.18 μM. A time-of-addition assay and transient-replicon assay were performed to study its mechanism of action and highlighted that itraconazole targets a step involved in RNA replication or polyprotein processing. Furthermore, resistance to itraconazole was associated with mutations in protein 3A [58].

#### 2.2.4. 3D^pol^ Inhibitors

Inhibitors of EV 3D^pol^ are classified into two major groups: (i) nucleoside-based inhibitors (NIs) and (ii) non-nucleoside-based inhibitors (NNIs) [11,59].

**Figure 16:**
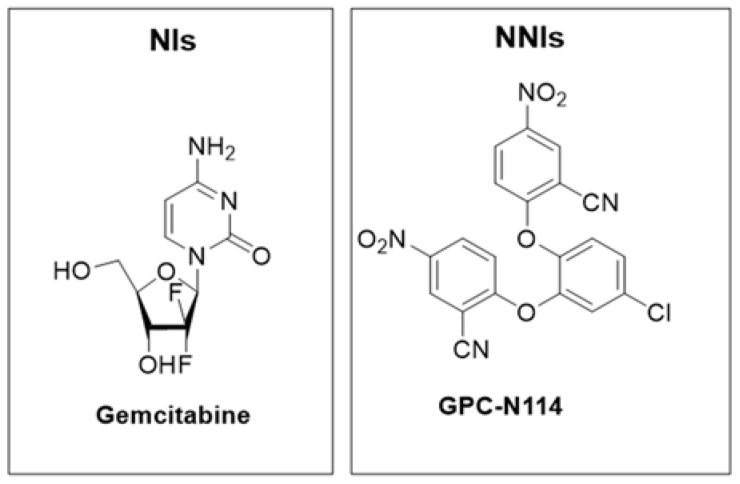
Chemical structures of gemcitabine and GPC-N114.

Gemcitabine (Figure 16), an FDA approved anticancer drug, was identified through the screening of 1280 bioactive compounds (LOPAC library) and showed a good inhibitory activity against CVB3 replication [60].

It was shown that gemcitabine has a dose-dependent antiviral activity [60]. The estimated IC_50_ in CVB3-infected HeLa cells was approximately 5 μM and cytotoxicity was observed at the highest concentration tested (50 μM). A time-of-addition assay revealed that gemcitabine targeted any step after viral entry into the host cells. The authors suggested that: (i) gemcitabine might be incorporated into enteroviral RNAs, thus causing mutations; and (ii) gemcitabine might directly interact with 3D^pol^, preventing the entry of the nucleotides needed for the polymerization process. The synergism of this compound in association with ribavirin, a well-known, broad-spectrum NI used for the treatment of several viral infections, was studied to better disclose gemcitabine’s potential antiviral activity. Although ribavirin shows a weak antiviral activity against CV-B3, an association of increasing concentrations of ribavirin and a fixed concentration of 0.4 μM of gemcitabine had a potent antiviral effect on CV-B3, reducing viral replication by up to 80%. The combination index value of co-treatment of 0.4 μM gemcitabine and increasing concentration of ribavirin was smaller than one, and this confirmed their synergism [60]. An additional effect of gemcitabine is the suppression of pyrimidine biosynthesis, which in turn stimulates innate immunity response through the activation of interferon stimulated genes (ISGs), a main antiviral mechanism in the early stages of infection [61].

Novel compounds with a common 5-nitro-2-phenoxybenzonitrile scaffold were identified to develop new NNIs and showed good antiviral activities [62]. This study led to the identification of GPC-N114 (Figure 16), the most selective and potent derivative of the series [62]. GPC-N114 showed an EC_50_ of 0.15 ± 0.02 µM in an in vitro assay of replication in a multicycle CPE-reduction of CVB3; instead, in a single cycle of CVB3 replication, the maximal inhibitory effect of this compound occurred at the concentration of 3 µM and higher. X-ray crystallographic studies showed that the complex with the first 2-cyano-4-nitrophenyl ring is exposed to the solvent, while the other occupies the entrance of the channel reserved for the template primer. Indeed, this compound occupies the nucleotide acceptor pocket, preventing any binding with the incoming nucleotide. As the structure of 3D^pol^ is highly conserved among the different EV serotypes, it was possible to compare the inhibitory mechanism of GPC-N114 against the 3D^pol^ of encephalomyocarditis virus (EMCV) and CVB3. In previous studies, EMCV resistance mutations suggested that GPC-N114 acts on 3D^pol^ by impairing the elongation of the new synthesized viral RNAs. In CVB3, the binding pocket of the antiviral compound is close to the mutated residues of 3D^pol^ in resistant EMCV. According to these data, GPC-N114 seems to overlap the binding site of the template primer of RNA, hence impairing the replication process [62].

### 2.3. Inhibitors of the Host Factors

Two different approaches can be considered to obtain broader antiviral drugs with a low mutation rate: one is to enhance the innate immune inflammatory response to boost host antiviral defense system [63,64,65], and another is to target host cellular factors essential for virus survival [61]. Given the fact that EVs can utilize the same host factors for cellular attachment, replication and assembling processes, host-targeting antivirals offer a new broad spectrum and a “resistance-proof” approach rather than pathogen-targeting antivirals, and have become a rising focus in EV research [11,66,67]. Numerous host factors play important roles in viral entry and replication [68]. Among these, proteins involved in the phosphatidylinositol 4-phosphate (PI4P)-OSBP pathway are important to enrich cholesterol during virus replication, while other essential factors in virus replication and assembling include glutathione [69,70,71,72], cyclophilins [73], heat shock proteins (HSPs) [74,75], Golgi-specific brefeldin A-resistance guanine nucleotide exchange factor 1 (GBF-1) [76,77,78], the host lipolysis pathway [79] and the DEAD-box polypeptide 3 (DDX3) [80]. Here, we focus on antivirals targeting the PI4KIIIβ-PI4P-OSBP pathway, which are the most advanced host factor inhibitors against EV-B infections.

#### Inhibitors of PI4KIIIβ-PI4P-OSBP Pathway

EVs reorganize host cellular endomembranes into replication organelles (ROs) to use them as platforms for viral replication [81]. During RO formation, several host factors needed for genome replication are recruited by enteroviral proteins. PI4KIIIβ is an enzyme involved in the production and trafficking of PI4P from the Golgi, while OSBP contributes to cholesterol and lipid homeostasis. Once recruited by the viral protein 3A, PI4KIIIβ generates PI4P, which is exchanged with cholesterol with the help of OSBP. The resulting cholesterol accumulation at the RO is essential for the formation of a viral replication complex needed for the synthesis of the viral positive-strand RNA [3,82].

**Figure 17:**
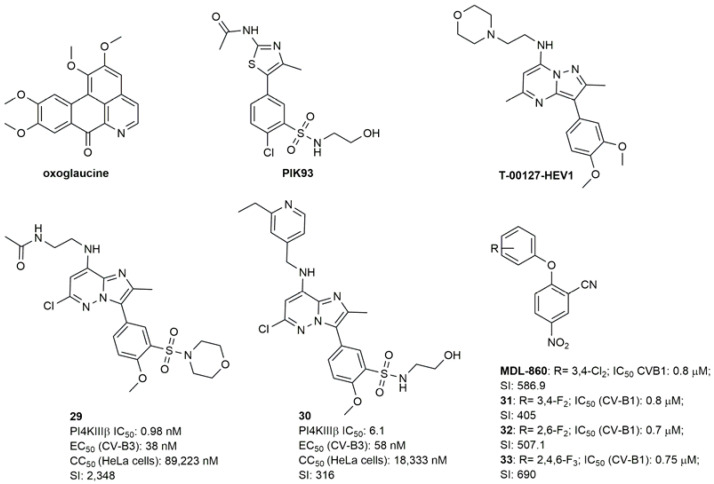
Chemical structures of the PI4KIIIβ inhibitors and biological activities of compounds **29**–**33**.

As above-mentioned, enviroxime (Figure 14) was identified as a potent antiviral compound against RV in the late 1970s. Only in 2012, it was shown that it directly inhibits PI4KIIIβ by reducing PI4P levels [83]. Enviroxime-like compounds have no structural similarities to enviroxime but exert the same effect on PI4KIIIβ. CV-B3 is able to acquire resistance to this class of inhibitors by acquiring a mutation at 3A, proving that viruses can develop resistance even if the target is a host-factor. They include oxoglaucine (Figure 17), TTP-307 (Figure 15), PIK93 and T-00127-HEV1 (Figure 17) [84]. Oxoglaucine (Figure 17) is one of the major enviroxime-like compounds inhibiting PI4KIIIβ, and is also in CV-B4, CV-B5 and CV-B3 [85]. The purine scaffold of T-00127-HEV1 has also been extensively studied [84,86]. Based on the observation that PIK93 and T-00127-HEV1 bind the ATP binding site of PI4KIIIβ in a similar manner, Mejdrovà et al. designed a second generation of hybrid compounds by combining chemical features of both parent compounds [87]. This strategy yielded a wide range of antivirals with excellent inhibitory activities and moderate to excellent selectivities toward the PI4KIIIβ enzyme. Compound **29** (Figure 17) showed high potency in enzymatic assays, but low in vitro permeability. Compound **30** (Figure 17) was then selected as hit, since it showed consistent nanomolar activity in cell-based assays against different viruses, including CV-B3.

The diarylether MDL-860, a broad spectrum antiviral developed in the ‘80s, is an atypical enviroxime-like compound which acts as an allosteric and irreversible covalent PI4KIIIβ inhibitor [88]. This scaffold has inspired the development of 12 analogues bearing the 2-cyano-4-nitrophenyl moiety bridged through N, S or O to different aromatic and aliphatic groups [89]. The substitution of the two chlorine atoms with two or three fluorine atoms (compounds **31**–**33**) retained similar potency and improved selectivity against CV-B1, but reduced activity against CV-B3. The introduction of other substituents, such as heterocyclic, alkyl and aryl groups did not lead to any significant improvement. Compound **31** was the most effective (50% protection effect) in experimental CVB1 neuroinfection in newborn mice infected with a high dose of virus inoculum (20 LD_50_) [90].

**Figure 18:**
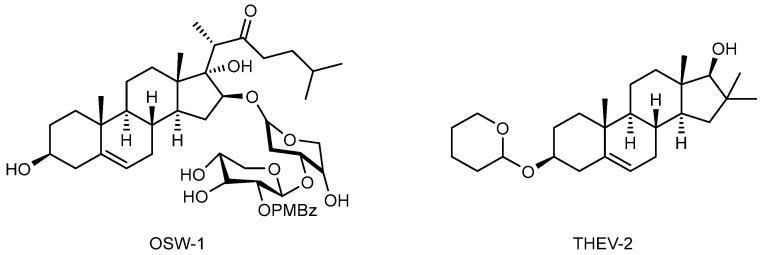
Chemical structures of the OSBP inhibitors OSW-1 and THEV-2.

The possibility of acquiring resistance to major enviroxime-like compounds through a mutation in 3A has underlined the importance of identifying inhibitors of the host factors acting on different targets. Four structurally diverse antivirals, also known as minor enviroxime-like compounds, hamper the formation of ROs by modulating OSBP with a different mode of actions: OSW-1 (Figure 18) [91], T-00127-HEV2 (Figure 18) [92], itraconazole [52] and TTP-8307 [57] (Figure 15).

Based on the results of Roberts et al., both OSW-1 and T-00127-HEV2 interact with the OSBP phospholipid binding site, whereas itraconazole and TTP-8307 have a different binding mode, likely in the OSBP C-terminal domain [93]. This suggests the possibility to co-administer OSW-1 and itraconazole, making them the two most pharmacologically relevant molecules of this class [93]. OSW-1 is an antiproliferative natural product compound that acts by altering ORP4 function, which is important for cell proliferation and viability. ORP4 is a close member of the family of the OSBP/ORP family and shares similar sequence with OSBP. It is reported that OSW-1 interacts with one of the two binding sites of OSBP (oxysterol or phospholipid binding sites). OSW-1 induced a reduction in cholesterol content in infected cells and showed antiviral activity against multiple EV strains, including EV-Bs. However, cytotoxicity is evident after a continual treatment with this compound, likely due to its interaction with ORP4 [94].

Itraconazole is a promiscuous anti-cancer and anti-fungal compound identified in different repurposing screenings as a broad-spectrum antiviral agent [95,96,97]. As mentioned in Section 2.2.3, itraconazole interferes with enteroviral replication independently from its anti-fungal and anti-cancer activities and is inactive against 3A-resistant mutants, suggesting 3A as a potential target. These mutations were located in a terminal and hydrophobic domain of 3A and did not confer resistance to other enviroxime-like compounds inhibiting PI4KIIIβ, indicating a different mode of action [58]. This clearly implies a link between 3A, itraconazole and PIP; indeed, PIP4 monitoring in CV-B3 infected treated and untreated cells revealed that PIP4 levels were higher compared to uninfected cells, ruling out PI4KIIIβ as a possible target. Instead, it was thoroughly demonstrated that itraconazole acts through the targets OSBP and ORP4 and inhibits OSBP lipid shuttling [52]. Bauer et al. analyzed the structural features essential for the OSBP-mediated activity through a combination of in silico and SAR studies. The triazole moiety, which is important for antifungal activity, can be replaced without affecting potency towards OSBP, while the sec-butyl chain tolerates only minor variations. This model not only suggests the importance of this moiety for the interaction in the binding pocket, but can also facilitate itraconazole optimization, allowing the design of less toxic itraconazole analogues more selective for OSBP [98]. Novel OSBP-selective inhibitors devoid of affinity for ORP4 are needed and could be powerful chemical probes to disclose OSBP biological function, fully reveal its therapeutic potential and develop a novel class of broad-spectrum antiviral inhibitors.

## 3. Conclusions

There is currently no specific treatment or prophylaxis against EV-B infections. The emergence of several outbreaks, as well as experimental evidence supporting the role of persistent CV infections in T1DM pathogenesis, have pointed to the need to find an optimal anti-viral treatment against these infections, which can be life-threatening in at-risk populations, such as children and the immunocompromised. As their clinical relevance has become more serious, efforts in the field of anti-EV-B inhibitors have significantly accelerated. Over the last decade, many potential antivirals with very high SIs and promising in vitro activities have been discovered, but despite great efforts, little success has been achieved and none of these experimental drugs has reached the market. The main challenges to anti-EV drug discovery are high mutation rate, lack of broad-spectrum effects, and failure of transition from in vitro studies to in vivo efficacy. The most advanced candidates in clinical trials are capsid binders, but their use is limited by the emergence of resistant strains and the narrow spectrum of activities. Many target-based antiviral screenings have therefore focused on non-structural proteins, which are more innovative targets for direct-acting broad-spectrum antivirals. Another valuable option to obtain broader antivirals with limited resistance in viruses is to target host factors, but this might lead to unwanted side effects, as the host protein could be essential for cellular functions. Indeed, several biological questions should be addressed and fundamental research on EV proteins and essential host factors is fundamental to fill the gap in infection dynamics and obtain broad-spectrum antivirals active against different EV serotypes.

Even though antiviral drug discovery for EV-A71 is more advanced compared to other EV species [99], in the last decade, drug discovery and medicinal chemistry efforts have provided many EV-B inhibitors with different mechanisms of action. Looking to the future, combining inhibitors with synergistic effects could be a strategy to both overcome drug resistance and broaden the spectrum of activity. Hopefully, such a combination therapy will translate into broad and effective antiviral treatments devoid of side effects and with potential clinical success.

## Data Availability

Data sharing is not applicable.
